# Is a cross-connector beneficial for single level traditional or cortical bone trajectory pedicle screw instrumentation?

**DOI:** 10.1371/journal.pone.0253076

**Published:** 2021-06-11

**Authors:** Frédéric Cornaz, Jonas Widmer, Marie-Rosa Fasser, Jess Gerrit Snedeker, Keitaro Matsukawa, José Miguel Spirig, Mazda Farshad

**Affiliations:** 1 Department of Orthopaedics, Balgrist University Hospital, Zurich, Switzerland; 2 Institute for Biomechanics, ETH Zurich, Zurich, Switzerland; 3 Department of Orthopaedic Surgery, National Hospital Organization, Murayama Medical Center, Musashimurayama, Tokyo, Japan; Rush University Medical Center, UNITED STATES

## Abstract

The cortical bone trajectory (CBT) has been introduced with the aim of better screw hold, however, screw-rod constructs with this trajectory might provide less rigidity in lateral bending (LB) and axial rotation (AR) compared to the constructs with the traditional trajectory (TT). Therefore, the addition of a horizontal cross-connector could be beneficial in counteracting this possible inferiority. The aim of this study was to compare the primary rigidity of TT with CBT screw-rod constructs and to quantify the effect of cross-connector-augmentation in both. Spines of four human cadavers (T9 –L5) were cropped into 15 functional spine units (FSU). Eight FSUs were instrumented with TT and seven FSUs with CBT pedicle screws. The segments were tested in six loading directions in three configurations: uninstrumented, instrumented with and without cross-connector. The motion between the cranial and caudal vertebra was recorded. The range of motion (ROM) between the CBT and the TT group did not differ significantly in either configuration. Cross-connector -augmentation did reduce the ROM in AR (16.3%, 0.27°, p = 0.02), LB (2.9%, 0.07°, p = 0.03) and flexion-extension FE (2.3%, 0.04°, p = 0.02) for the TT group and in AR (20.6%, 0.31°, p = 0.01) for the CBT-group. The primary rigidity of TT and CBT single level screw-rod constructs did not show significant difference. The minimal reduction of ROM due to cross-connector-augmentation seems clinically not relevant. Based on the findings of these study there is no increased necessity to use a cross-connector in a CBT-construct.

## Introduction

**Fig 1:**
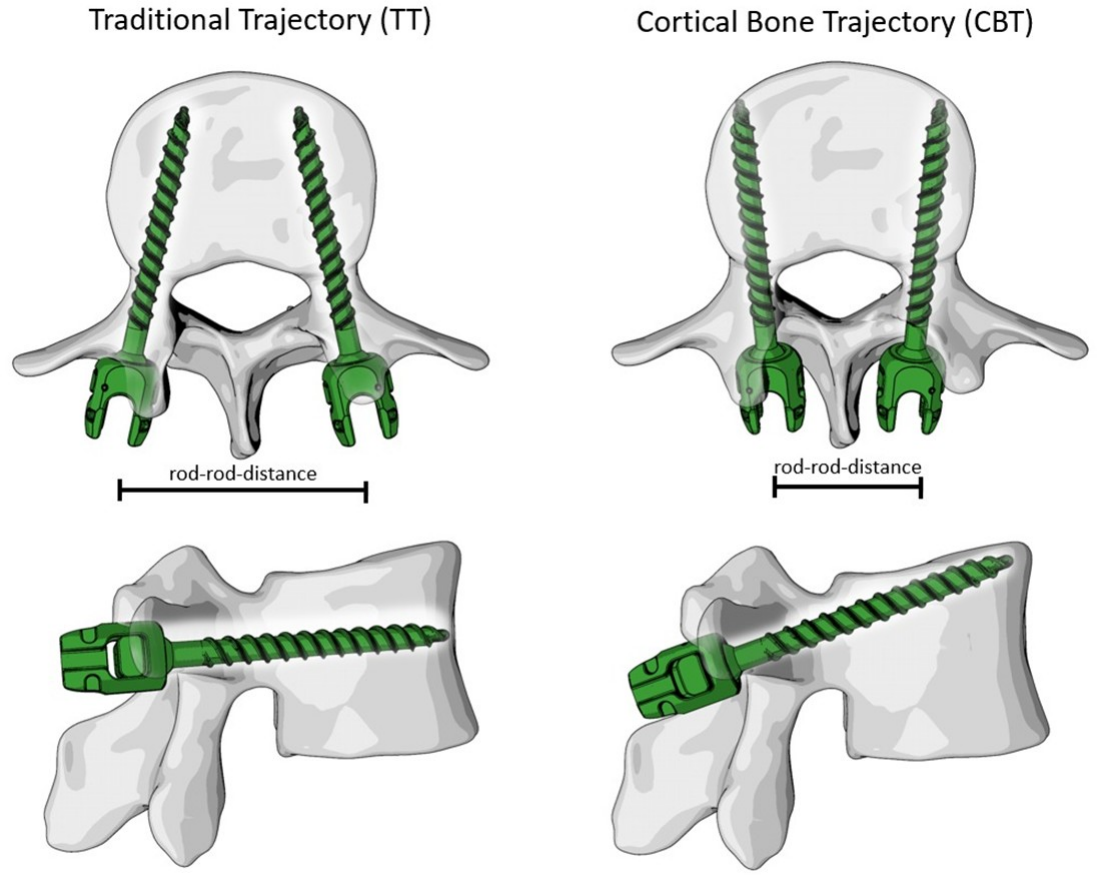
Screw trajectories. Schematic representation of a vertebral body instrumented with pedicle screws following the traditional trajectory (left) and the cortical bone trajectory (right). Note the smaller distance between the screw heads and the more sagittal orientation of the cortical bone trajectory configuration. Figure generated by the authors.

Dorsal pedicle screw instrumentation of the spine is an effective method to attain primary stability required for bony fusion. More rigid instrumentations lead to higher fusion rates [1] and consequently, instrumentations for fusion are optimized to provide high stiffness in all loading directions. Pedicle screws used for this purpose are mostly implanted in the traditional trajectory (TT) following the anatomical axis of the pedicle. In this lateral-to-medial trajectory, most of the screw thread is located in cancellous bone which has been shown to be disadvantageous for patients with inferior bone quality such as in osteoporosis [2]. As a consequence, Santoni et al. [3] proposed the cortical bone trajectory (CBT) with an entry point placed more medially and a trajectory in a caudocephalad and mediolateral direction. This orientation is intended to increase the contact area of the screw thread with cortical bone and thereby increase anchoring strength in comparison to TT. Superior pullout-strength [3] and higher resistance in craniocaudal toggling tests [4] were measured for CBT pedicle screws in comparison to TT pedicle screws in human cadavers. With the entry points being placed more medially, CBT constructs generally have a smaller posterior rod-rod distance (Fig 1).

Intuitively, this raises the question whether these narrower constructs provide less shear stability which could be disadvantageous in construct stiffness, particularly in axial rotation (AR), lateral bending (LB) and lateral shear (LS). Vice-versa CBT screws are aligned more sagittal which could be beneficial in flexion-extension (FE) (Fig 1). Supporting these considerations, a recent finite element study found CBT-constructs to be less stiff in AR and LB, but stiffer in FE in comparison to TT constructs [5]. Additionally, in a cadaver study, CBT screw-rod constructs did show a non-significant trend towards larger ROM in AR and LB in comparison to TT constructs [6].

The addition of horizontal cross-connectors (CC) to TT screw-rod constructs has been demonstrated to increase rigidity of dorsal instrumentations in AR and LB [7–12]. With the possible inferior stability of CBT-constructs in AR and LB, some surgeons argue the necessity and effectiveness of CC-augmentation in CBT constructs. While some limited evidence can be found for the beneficial effect of CC on TT-constructs [7–12], none is available for CBT constructs.

These considerations lead to three hypotheses: 1) CBT-constructs are less stable in AR, LB and LS, 2) CBT constructs are more stable in FE and 3) CC-augmentation is more effective in CBT-constructs compared to TT-constructs. Therefore, the aim of this study was to compare the primary rigidity of single level CBT-constructs to TT-constructs and to quantify the effect of CC-augmentation on both trajectories.

## Materials and methods

### Dissection, preparation and storage

Four fresh frozen cadavers of human donors (Science Care, Phoenix, AZ, USA) were used for this study (Table 1). Ethical approval was obtained by the local authorities (Swissethics, BASEC Nr. 2017–00874). The original written informed consent for donation, in accordance with applicable law and regulation and content for cremation, are on file at the offices of Science Care. All medical information was fully anonymized. The cadavers (accessed June 2018) were stored at– 20°C until dissection and instrumentation. CT scans of all specimens were taken to exclude bony defects and spinal deformities and severe degeneration. The Th9/Th10 segment of one specimen was therefore excluded due to intervertebral disc ossification. The CT data was also used to quantify the bone density of the vertebral bodies [13]. Alternating between TT and CBT to generate equivalent groups, the trajectories were planned by an experienced spine surgeon using the MySpine software based on the CT-data (Medacta International, Castel San Peitro, Switzerland) [14]. Screw diameter and length were maximized as recommended [15]. After thawing, the specimens were carefully dissected without harming paraspinal ligaments and the intervertebral discs. The specific 2.7 mm drill guides generated by the planning software were 3D-printed and used for instrumentation of cannulated poly-axial pedicle screws (ref. 03.52.3xx, M.U.S.T, Medacta International, Switzerland). The dorsal aspects of the spinous processes were removed to provide space for later mounting of CC. After instrumentation, the segments were mounted with 3D-printed-clamps for biomechanical testing [16].

**Table 1:** Specimen overview.

		Instrumented levels	Trajectory	Cranial left screw [mm]	Cranial right screw [mm]	Caudal left screw [mm]	Caudal right screw [mm]	CT-value [HU]
Age / gender	65 y / female	Th10/Th11	TT	45x6	45x6	45x6	45x6	132
Height	162 cm	Th12/L1	CBT	40x5	40x5	40x5	40x5	122
weight	68 kg	L2/L3	TT	45x6	45x6	45x6	45x6	109
BMI	25	L4/L5	CBT	45x6	45x6	40x6	40x6	105
Age / gender	45 y / female	Th10/Th11	CBT	40x5	40x5	40x5	40x5	128
Height	165 cm	Th12/L1	TT	45x4.5	45x4.5	45x4.5	45x4.5	134
weight	59 kg	L2/L3	CBT	40x4.5	40x4.5	40x4.5	40x4.5	103
BMI	21.6	L4/L5	TT	50x6	50x6	50x6	50x6	110
Age / gender	62 y / male	Th09/Th10	CBT	40x5	40x5	40x5	40x5	130
Height	167 cm	Th11/Th12	TT	45x6	45x6	45x6	45x6	156
weight	54 kg	L1/L2	CBT	40x5	40x5	45x5	45x5	147
BMI	19.4	L3/L4	TT	55x6	55x6	50x6	50x6	127
Age / gender	64 y / male	Th09/Th10	TT	excluded	excluded	excluded	excluded	excluded
Height	180 cm	Th11/Th12	CBT	40x5	40x5	45x6	45x6	51
weight	117 kg	L1/L2	TT	55x6	55x6	55x6	55x6	107
BMI	36.1	L3/L4	CBT	45x6	45x6	40x6	40x6	120

Overview of the specimens used for the experiments. Information on the donors, the instrumented levels, the trajectories and the implanted pedicle screw size as well as the CT-value averaged for the two vertebral bodies are stated. TT = traditional trajectory, CBT = cortical bone trajectory

### Biomechanical testing

**Fig 2:**
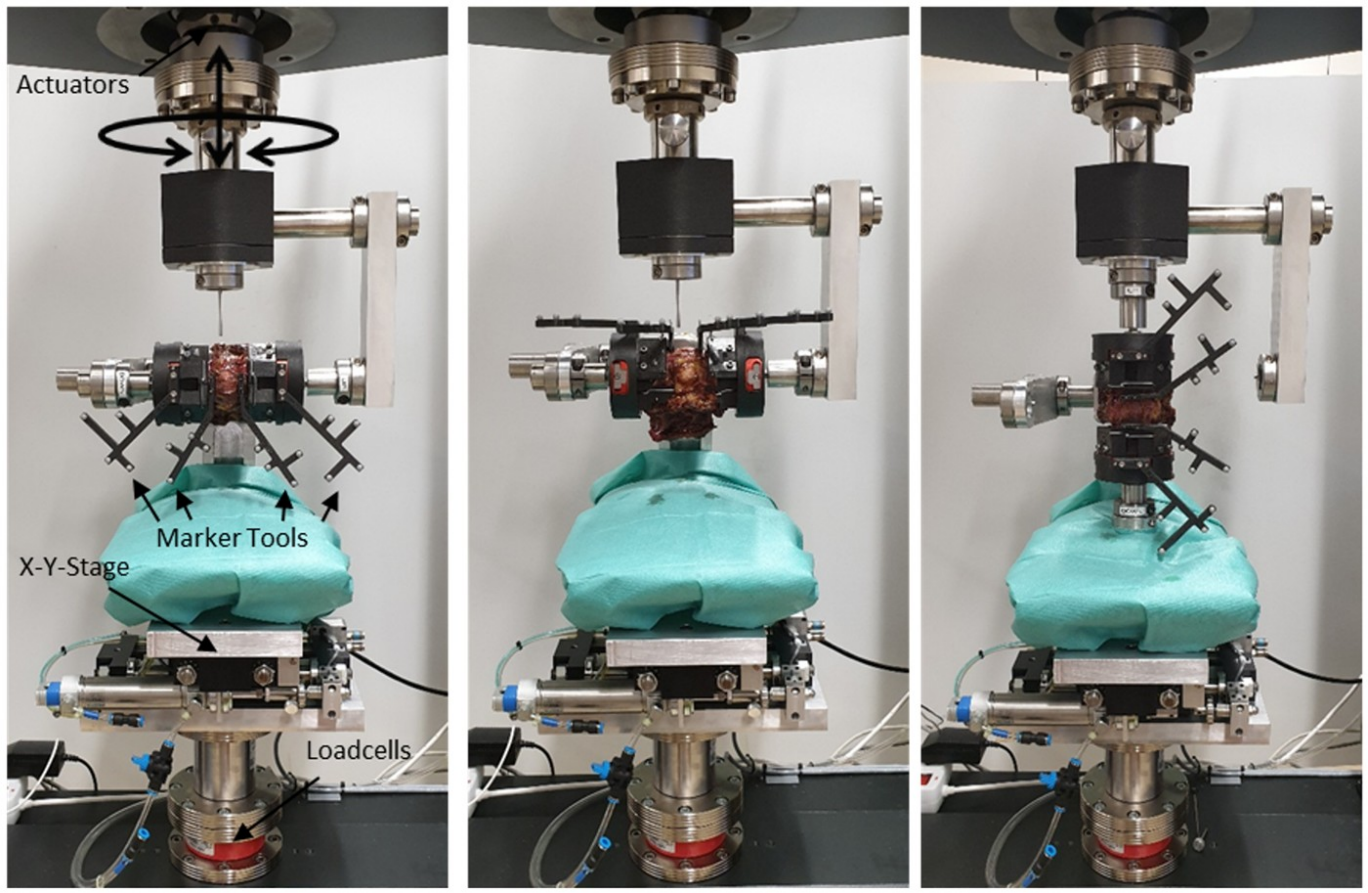
Mechanical test setup. The setup used for biomechanical testing can be used for flexion-extension and lateral shear (left), lateral bending and anteroposterior shear (center) and axial rotation and axial compression-decompression (right). Figure adapted from [18].

Biomechanical testing was performed on a biaxial linear-torsion static testing system (Zwick/Roell Allroundline 10kN and testXpert III Software, ZwickRoell GmbH & Co. KG, Germany). The machine consists of a traverse to generate compression and tension along the z-axis and a torsion motor to generate torque around the z-axis. This machine was supplemented with a setup consisting of an x-y-table and holding arms that allow for specimen fixation in a vertical orientation for axial compression-decompression (AC) and axial rotation (AR) and in a horizontal orientation for lateral bending (LB) and anteroposterior shear (AS) as well as flexion-extension (FE) and lateral shear (LS) (Fig 2). FE, LB and AR were applied with 1°/sec and torque of ± 7.5 Nm. AS and LS were applied with 0.5 mm/sec to ± 150 N. AC was applied with 0.1 mm/sec to 400 N compression and—150 N decompression. The torques and forces conform to commonly used values in the literature [17]. Loading was applied to the cranial vertebra while the caudal vertebra was fixed to the test rig with the x-y-table allowing for translational movement orthogonal to the loading direction. Coupled motion around the x- and y-axis were prevented, restricting all movement to the testing plane. With this configuration, translational forces—as they could occur in a fully constrained setup—are omitted resulting in pure moments and pure forces in the plane of interest.

**Fig 3:**
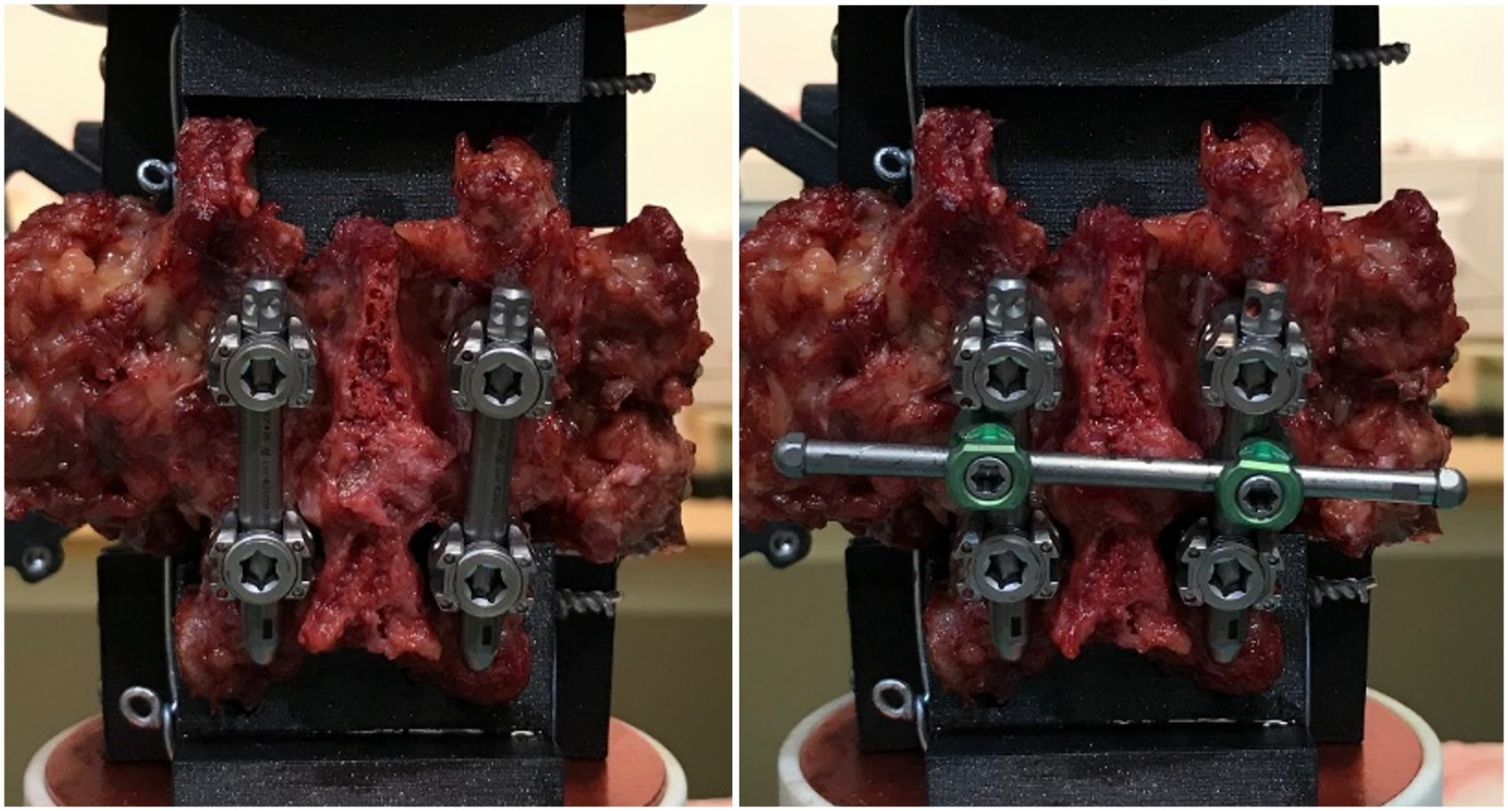
Cross-connector. CBT screw-rod construct without horizontal cross-connector (left) and augmented with one horizontal cross-connector (right). Figure generated by the authors.

After five preconditioning cycles, the movement of the cranial and caudal vertebral body was recorded over one cycle with a motion capturing system (Atracsys Fusion Track 500, 10 Hz record frequency, tracking accuracy 0.09 mm (RMS)). The segments were frequently sprayed with phosphate buffered saline (PBS) to prevent tissue dehydration. The loading protocol was repeated for all six loading directions. Every FSU was tested under three conditions—1) uninstrumented, 2) instrumented and 3) instrumented with horizontal CC, whereby the test sequence of 2) and 3) was reversed for half of the samples to prevent potential bias. Uninstrumented testing was performed with implanted pedicle screws, but without vertical rods. For instrumented testing, pedicle screws were interlinked with vertical rods (pre-bent rods, titanium 5.5x50 mm, ref. 03.50.453, M.U.S.T, Medacta International, Switzerland) on either sides and set screws were tied with 9 Nm torque according to the standard surgical technique. One CC (straight cross connector, ref. 03.56.408, M.U.S.T Medacta International, Switzerland) was mounted horizontally in the center of the two rods and locked with 5.5 Nm torque according to the standard surgical technique (Fig 3). A camera with a telecentric objective (Edmund Optics #62–921, 182mm WD, 0.28X, Edmund Optics Inc., Barrington, NJ, USA) was used to image the screw rod construct in the neutral position to accurately measure the rod-rod distance. The total testing time added up to maximal 2h per FSU.

### Statistical evaluation

**Fig 4:**
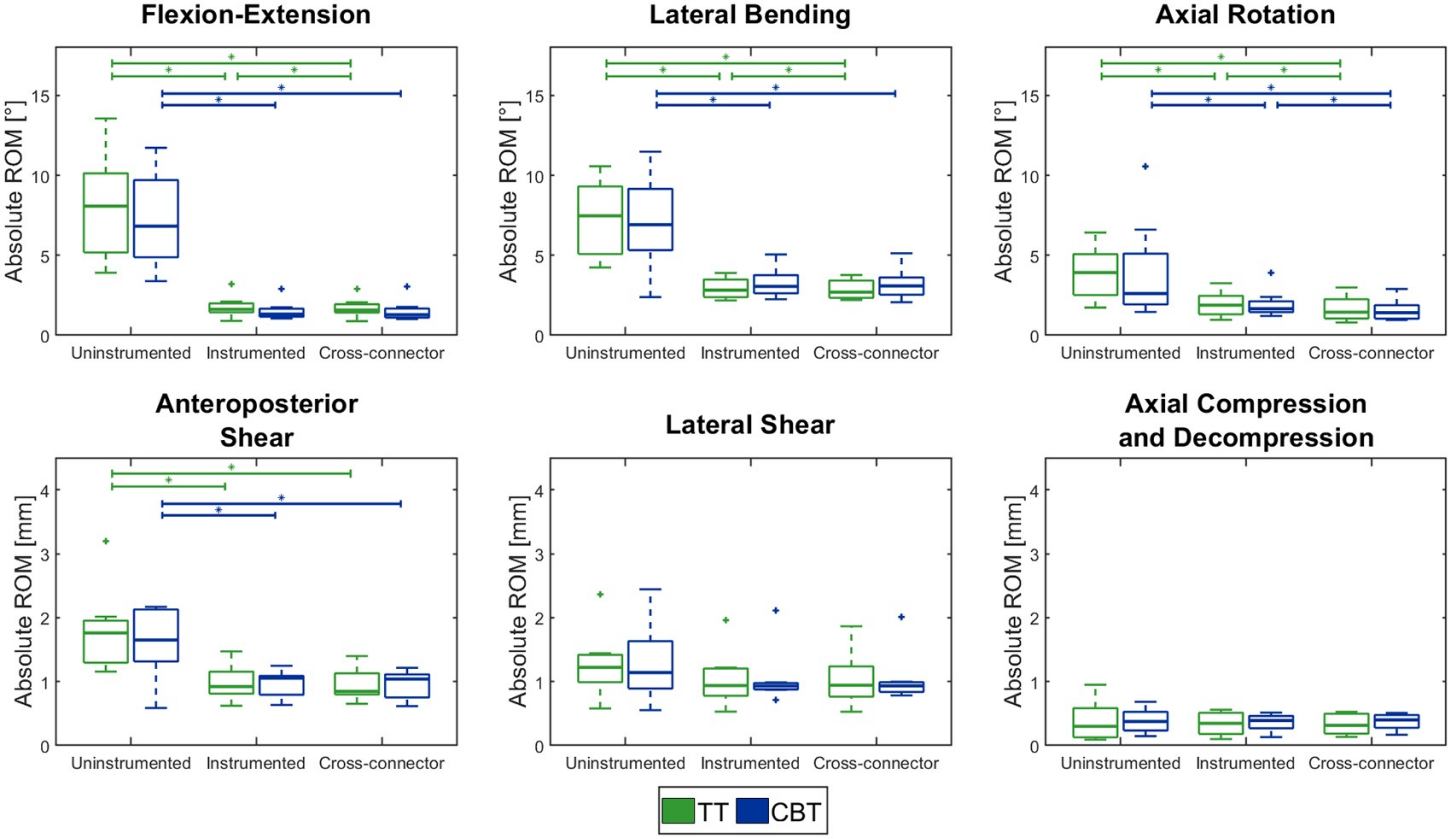
Range of motion. Boxplots for the range of motion (ROM) for uninstrumented, instrumented and cross-connector configuration between TT (green, left) and CBT (blue, right) shown for flexion-extension, lateral bending, axial rotation, anteroposterior shear, lateral shear and axial compression/decompression. Significant differences between the configurations are marked with asterisks (p<0.05), TT = traditional trajectory, CBT = cortical bone trajectory. Figure generated by the authors.

**Fig 5:**
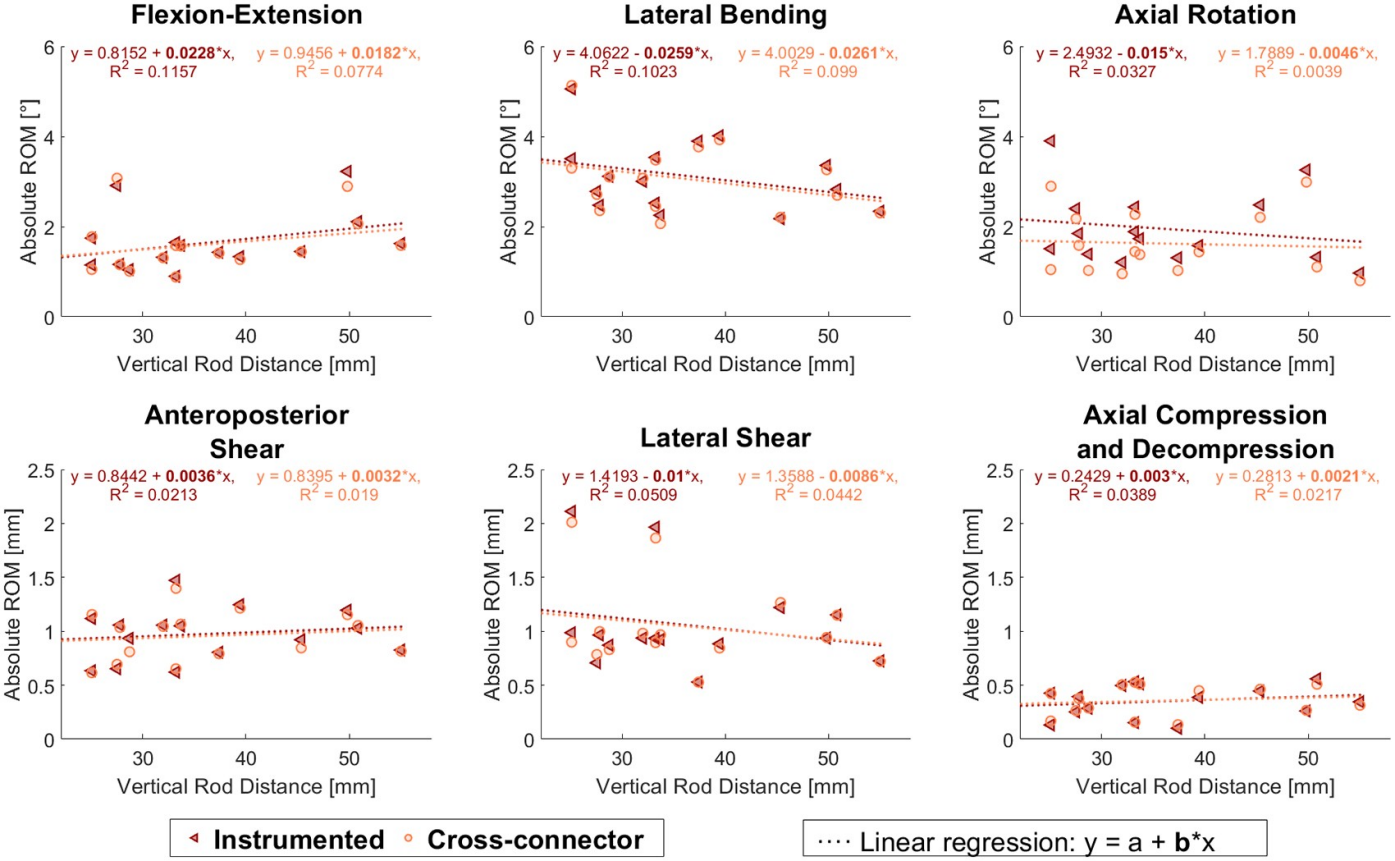
Rod-rod distance. Scatter plot for the range of motion (ROM) over the rod-rod distance for instrumented (triangles) and cross-connector-configuration (circles) shown for all six loading directions. No statistically significant correlations were observed. Linear regression fit and their parameters are reported to illustrate the data distribution. Figure generated by the authors.

Screw-length, screw-diameter and bone density of the two groups were normally distributed and thus were compared using the two-sample t-test. Motion tracking data was used to calculate the ROM over the whole loading amplitude. For translational directions (LS, AS, AC), the relative translational movement of the markers was assessed. For rotational movements (FE, LB, AR), the projected angular movement in the motion plane was evaluated. Shapiro-Wilk parametric hypothesis tests of composite normality showed not all residuals of the ROM and reduction values to be normally distributed. Thus, the Mann-Whitney U test was used to evaluate the difference between the two trajectory groups and the Wilcoxon signed-rank test for paired data was used to compare the effect of the instrumentations on the ROM (Fig 4). The significance level was defined at α = 0.05. The relationship between ROM and rod-rod distance was evaluated with a spearman correlation test. Furthermore, a linear regression fit was performed on these data (Fig 5).

## Results

The specimens of the two groups did not differ significantly in CT-values with 125 ± 17.8 HU (mean ± SD) for TT and 113 ± 28.8 HU for CBT (p = 0.37). The ROM in the uninstrumented configuration of the TT and CBT group did not differ significantly (p>0.05) (Fig 4, Table 2). The pedicle screws used for the TT group were longer (TT 48.2 ± 4.1 mm, CBT 41.3 ± 2.2 mm, p<0.001) and thicker (TT 5.8 ± 0.5 mm, CBT 5.3 ± 0.5 mm, p<0.001). In both groups, the instrumentations with and without CC reduced ROM significantly for AR, FE, LB and AS compared to the uninstrumented configuration (p<0.05) (Fig 4, Table 2). Neither in the instrumented, nor in the cross-connector configuration did the ROM differ significantly between the two trajectory groups (p>0.05). There was however a trend towards smaller ROM in FE and larger ROM in LB for the CBT constructs (Fig 4, Table 2). CC-augmentation lead to a significant reduction in ROM compared to the instrumented configuration without CC in AR (16.3%, 0.27°, p = 0.02), LB (2.9%, 0.07°, p = 0.03) and FE (2.3%, 0.04°, p = 0.02) for the TT group and in AR (20.6%, 0.31°, p = 0.01) for the CBT-group (Fig 4, Table 3).

**Table 2:** Recorded range of motion.

		FE [°]	LB [°]	AR [°]	AS [mm]	LS [mm]	AC [mm]
**TT**	**Uninstrumented**	**8.1** (5.1, 10.1)	**7.5** (5.1, 9.3)	**3.9** (2.5, 5.1)	**1.8** (1.3, 2.0)	**1.2** (1.0, 1.4)	**0.3** (0.1, 0.6)
	**Instrumented**	**1.6** (1.4, 2.0)	**2.8** (2.4, 3.5)	**1.9** (1.3, 2.5)	**0.9** (0.8, 1.2)	**0.9** (0.8, 1.2)	**0.3** (0.2, 0.5)
	**cross-connector**	**1.6** (1.4, 1.9)	**2.7** (2.3, 3.4)	**1.4** (1.0, 2.3)	**0.9** (0.8, 1.1)	**0.9** (0.8, 1.2)	**0.3** (0.2, 0.5)
**CBT**	**Uninstrumented**	**6.8** (4.9, 9.7)	**6.9** (5.3, 9.2)	**2.6** (1.9, 5.1)	**1.7** (1.3, 2.1)	**1.1** (0.9, 1.6)	**0.4** (0.2, 0.5)
	**Instrumented**	**1.3** (1.2, 1.7)	**3.1** (2.6, 3.8)	**1.7** (1.4, 2.1)	**1.1** (0.8, 1.1)	**0.9** (0.9, 1.0)	**0.4** (0.3, 0.5)
	**cross-connector**	**1.3** (1.1, 1.7)	**3.1** (2.5, 3.6)	**1.4** (1.0, 1.9)	**1.0** (0.8, 1.1)	**0.9** (0.8, 1.0)	**0.4** (0.3, 0.5)

Median (25^th^ perc., 75^th^ perc.) values [°], [mm] of the range of motion for the TT and CBT group in the uninstrumented, the instrumented and the cross-connector configuration in all load directions. FE = flexion-extension, LB = lateral bending, AR = axial rotation, AS = anteroposterior shear, LS = lateral shear, AC = axial compression-decompression.

**Table 3:** Reduction in range of motion due to the addition of one horizontal cross-connector.

	TT		CBT	
	**[°]**	**[%]**	**[°]**	**[%]**
Flexion—Extension	**0.04** (0.02, 0.06), p = 0.02	**2.3** (1.4, 3.7)	**0.01** (0.01, 0.05), p = 0.05	**0.8** (-0.6, 4.2)
Lateral Bending	**0.07** (0.02, 0.12), p = 0.03	**2.9** (1.6, 3.2)	**0.08** (-0.03, 0.16), p = 0.15	**2.3** (-0.6, 5.3)
Axial Rotation	**0.27** (0.18, 0.28), p = 0.02	**16.3** (9.0, 20.4)	**0.31** (0.24, 0.41), p = 0.01	**20.6** (11.8, 25.9)
	**[mm]**	**[%]**	**[mm]**	**[%]**
Anteroposterior Shear	**0.01** (-0.02, 0.07), p = 0.3	**1.6** (-1.5, 4.6)	**0.02** (-0.03, 0.03), p = 0.7	**1.6** (-2.5, 2.7)
Lateral Shear	**0.00** (0.00, 0.03), p = 0.5	**0.3** (-0.4, 3.4)	**0.00** (-0.05, 0.06), p = 0.8	**0.5** (-4.8, 4.8)
Axial Comp. and Decomp.	**-0.01** (-0.01, 0.03), p = 1	**-2.1** (-3.5, 7.0)	**-0.01** (-0.02, 0.00), p = 0.3	**-1.2** (-10.3, 0.6)

Median (25^th^ perc., 75^th^ perc.) reduction of range of motion due to cross-connector addition in absolute [°], [mm] and percental [%] values for all loading directions in the TT and CBT group. Positive values indicate reduction of range of motion. Significant values are underlined (p<0.05). TT = traditional trajectory, CBT = cortical bone trajectory

No significant correlation was observed between the rod-rod distance and the ROM (p>0.05) (Fig 5). However, samples with smaller rod-rod distances showed a trend of larger ROM in LB, AR and LS. CC-augmentation in AR showed a tendency of being more effective for samples with smaller rod-rod distances (Fig 5). The slopes of the linear regression fit were below 0.03°/mm for torsion loads and below 0.01 mm/mm for translational loads (Fig 5).

## Discussion

Pedicle screws following the cortical bone trajectory have been demonstrated to be a valid alternative to TT pedicle screws in multiple aspects [19]. There are however certain indications of inferior stability in AR and LB and superior stability FE for CBT-constructs [5, 6]. These observations lead to hypotheses 1) CBT-constructs are less stable in AR, LB and LS and 2) CBT constructs are more stable in FE. In the here reported experiments, the range of motion of the two trajectory-groups did not differ in a statistically significant way. However, a trend towards larger ROM in LB and smaller ROM in FE for the CBT-group were observed (Fig 4, Table 3). Additionally, samples with smaller rod-rod distances showed a trend of larger ROM in AR, LB and LS (Fig 5). These findings partly support the theoretical considerations of the two hypotheses; however, these effects were statistically non-significant and with the small differences, we evaluate them as clinically non-relevant.

While CC-augmentation has been observed to increase rigidity of TT-constructs in AR and LB [7–12], no such information is available for CBT-constructs. The results of this study did show a statistically significant reduction in ROM in AR for both TT (16.3%, p = 0.02) and CBT (20.6%, p = 0.01) and in LB (2.9%, p = 0.03) as well as FE for TT (2.3%, p = 0.02). Nevertheless, in absolute values, the reduction was very small with 0.27° for TT and 0.31° for CBT in AR, only 0.04° for TT in FE and 0.07° for TT in LB (Table 3). Therefore, we believe this reduction to be non-relevant for surgical outcome and does not obligate the use of CC in single-level dorsal instrumentation of neither TT nor CBT constructs. The hypothesis 3) CC-augmentation is more effective in CBT-constructs compared to TT-constructs, was not confirmed, but there was a trend in AR with larger ROM-reductions in instrumentations with smaller rod-rod distances (Fig 5). We believe this tendency to be of minor importance, since it is not detectable while comparing the two trajectories.

The presented results provide further evidence that CBT constructs are associated with equivalent primary stability compared to TT constructs. This information is crucial for the clinical practice as it prevents unnecessary cross-connector-augmentation, which is related to additional implant cost and surgical time and has been associated with soft tissue irritation and delayed infection [20] and even with an increased risk for pseudarthrosis [21].

There are some limitations to this study. The study investigates the primary, passive rigidity in the six major axes in well defined, isolated load applications. However, in reality an infinite number of complex combined loading situations occurs [22]. The effectiveness of CC-augmentation as well as the stability of TT- and CBT-constructs could differ in such combined loading situations. Additionally, all experiments were conducted on single level instrumentations and conclusions for multilevel constructs are therefore limited. To evaluate only the effectiveness of the different configurations without additional factors, interventions such as decompression surgery or intervertebral cage implantation as well as the infliction of fractures or discoligamentous injuries were not performed. In situations with such interventions or injuries, the primary stability of the two trajectories as well as the effectiveness of CC-augmentation could be different, however we evaluate the general trends of the reported results to be similar.

## Conclusions

We conclude that the rigidity of lumbar and lower thoracic CBT-constructs is not significantly different to that of TT-constructs. Furthermore, our findings indicate that cross-connector-augmentation does not increase the rigidity of CBT or TT instrumentations in a clinically relevant way.

## Supporting information

S1 File The post-processed data including statistical analysis are combined in the supporting information file “All_Results.xls”. (ZIP)
